# Preclinical Evaluation of the Safety of the Dedicated Gel for Gel Immersion Endoscopy With Gastrointestinal Perforation and Peritoneal Leakage

**DOI:** 10.1002/deo2.70258

**Published:** 2025-12-08

**Authors:** Atsushi Ohata, Ryo Kawahara, Yuji Hiraki, Hikaru Nakata, Shinya Kaneda, Tomonori Yano

**Affiliations:** ^1^ Naruto Research Institute Research and Development Center Otsuka Pharmaceutical Factory Inc. Tokushima Japan; ^2^ Department of Medicine Division of Gastroenterology Jichi Medical University Tochigi Japan

**Keywords:** endoscopy, gels, immersion, safety, intestinal perforation

## Abstract

**Objectives:**

Gel immersion endoscopic mucosal resection or endoscopic submucosal dissection has been developed as an alternative to saline immersion therapeutic endoscopy. However, in cases of gastrointestinal perforation, gel may leak from the lumen into the peritoneal cavity, and the safety of such events remains unclarified. This study aimed to evaluate the safety of the dedicated gel (Viscoclear, VC) in the event of perforation and peritoneal leakage.

**Methods:**

A perforation model was created to compare the colonic burst pressure after VC or saline injection. Acute toxicity was assessed by intraperitoneal VC administration in rats at a maximum dose of 20 mL/kg. *Escherichia coli* was cultured for 24 h in VC or saline, and bacterial growth was evaluated. Cytotoxicity (colony formation of V79 cells), sensitization (guinea pig maximization test), and intracutaneous reactivity (local irritation in rabbits) were also examined.

**Results:**

The burst pressure was significantly higher for VC injection than for saline. There were no abnormalities indicative of toxicity observed in the general condition or at necropsy after VC administration, and the hematological and biochemical parameters were within normal limits. VC did not promote bacterial growth compared with saline. VC was not cytotoxic and did not cause skin sensitization. Intradermal reactivity tests showed that VC caused negligible irritation that was judged “acceptable”.

**Conclusions:**

These results suggest that VC is less likely to leak than saline and that, even in the event of a minor leakage into the peritoneal cavity, VC has no acute systemic toxicity and acceptable biocompatibility.

## 1 Introduction

Saline immersion therapeutic endoscopy (SITE) [1], including underwater endoscopic mucosal resection (EMR) and endoscopic submucosal dissection (ESD) [2, 3], has various beneficial effects, such as the buoyancy effect and heat‐sink effect. However, SITE has limitations, including loss of the visual field during arterial bleeding due to the rapid mixing of saline and blood, and the ineffectiveness of monopolar hemostatic forceps in saline because of electrical dissipation [4].

Gel immersion endoscopy has been reported as a useful method that secures the visual field during gastrointestinal bleeding [5]. The dedicated gel used in gel immersion endoscopy, Viscoclear (VC), is electrolyte‐free and has an appropriate viscosity for preventing rapid mixing with fresh blood or feces. The space occupied by the injected VC provides a clear visual field and working space for endoscopic procedures [6]. Gel immersion EMR and ESD have recently been developed as alternatives to SITE that facilitate easy and safe procedures [7, 8, 9, 10, 11, 12]. Furthermore, the gel tends to remain around the lesions longer than saline, which may be useful for superficial non‐ampullary duodenal epithelial tumors in such areas where saline immersion is difficult [9, 13].

**FIGURE 1:**
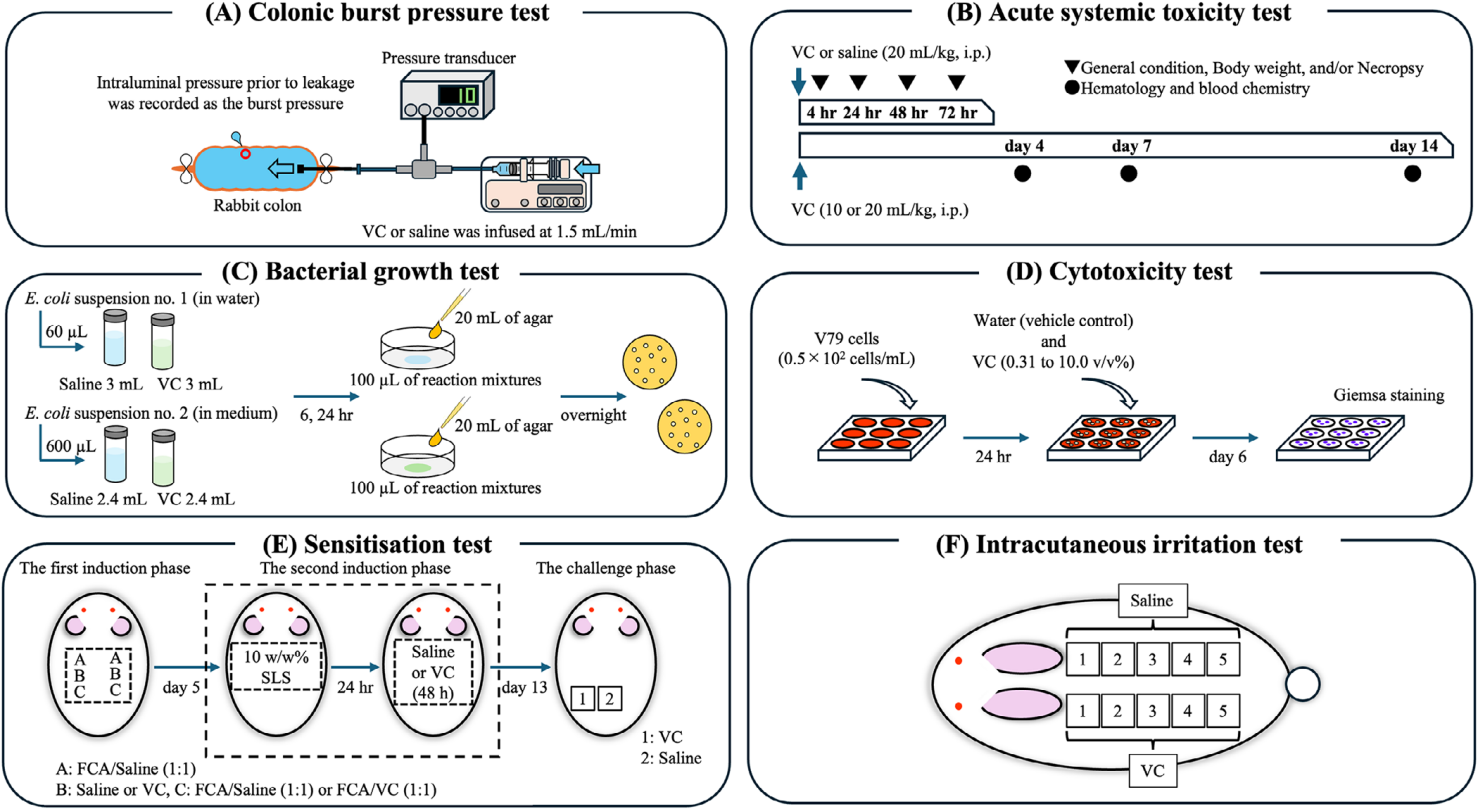
An overview of the experiments performed to evaluate the safety of VC. VC, Viscoclear (A) Colonic burst pressure test: Fresh ex vivo colonic segments from rabbits were perforated and infused intraluminally with either VC or saline at 1.5 mL/min until leakage occurred; the maximum pressure before leakage was recorded as burst pressure. (B) Acute systemic toxicity test: Rats received a single intraperitoneal dose of VC or saline (10 or 20 mL/kg), and general condition, body weight, necropsy, hematology, and biochemistry were evaluated. (C) Bacterial growth: *E. coli* suspensions were incubated with VC or saline under nutrient‐rich or nutrient‐free conditions for 6 or 24 h, and viable counts and growth rates were determined. (D) Cytotoxicity test: V79 cells were exposed to VC (0.31%–10% v/v) for 6 days; colony formation rate and IC_50_ were determined. (E) Sensitization test: Guinea pigs underwent intradermal induction and topical challenge with VC or saline; skin reactions were scored using the Magnusson–Kligman scale. (F) Intracutaneous irritation test: VC or saline was injected intracutaneously into rabbits, and erythema/eschar and edema were scored at 24, 48, and 72 h.

One of the severe adverse events associated with EMR and ESD is gastrointestinal perforation [14, 15, 16, 17]. VC is clearly labeled as not to be used in patients with confirmed or suspected gastrointestinal perforation. However, in clinical use, perforation may unintentionally occur during gel immersion EMR or ESD, resulting in leakage of the gel from the lumen into the peritoneal cavity. To the best of our knowledge, no study has systematically evaluated the biological safety of VC, and the safety of its leakage into the peritoneal cavity remains unexamined. Although gastrointestinal perforations are relatively rare, they are recognized as severe adverse events during endoscopic procedures; therefore, addressing this knowledge gap holds significant clinical and practical importance. In the present study, we evaluated the safety of VC. First, we evaluated the tendency of VC to leak from the lumen using a perforation model. Second, we assessed acute systemic toxicity after direct intraperitoneal administration of VC in rats. Third, we evaluated whether VC serves as a bacterial growth substance. Finally, we assessed the cytotoxicity, sensitization, and intradermal reactivity of VC in accordance with the criteria for the biological safety evaluation of medical devices [18]. An overview of the experimental procedures used in both the in vitro and in vivo studies is illustrated in Figure 1.

## 2 Materials and Methods

### 2.1 Test Article

VC (Otsuka Pharmaceutical Factory, Tokushima, Japan) was used as the dedicated gel for the experiments. VC (200 g/package) consists of 0.075 w/w% thickening polysaccharides (xanthan gum and locust bean gum), 2.4 w/w% concentrated glycerin (as an isotonic agent), and 97.525 w/w% purified water. In the present study, the gel used had an osmotic pressure ratio of 0.93, a pH of 5.7, a complex shear viscosity of 1037.2 mPa·s, and a shear storage modulus of 3.25 Pa.

### 2.2 Animals

All animal experiments and procedures were approved by the Committee on the Care and Use of Laboratory Animals, Otsuka Pharmaceutical Factory Incorporated, and were in full compliance with Japanese regulations. Male Crl:CD (Sprague Dawley) rats (Jackson Laboratory Japan, Inc., Kanagawa, Japan), female Slc:Hartley guinea pigs (Japan SLC, Shizuoka, Japan), and male Japanese White rabbits (Std:JW/CSK; clean animals; hair clipped before shipment; Japan SLC, Shizuoka, Japan) were housed under laboratory conditions (temperature, 20.0–26.0°C; humidity, 40.0%–75.0%; 12‐h light/dark cycle) for at least 1 week before the experiments and were fed a standard laboratory diet and provided with water ad libitum.

### 2.3 Colonic Burst Pressure Test

Fresh ex vivo colons were harvested from five euthanized rabbits. Each colon specimen measured approximately 15 cm in length with an internal diameter of 5–8 mm and was divided into four segments; therefore, a total of 20 colonic segments were analyzed. A full‐thickness defect was created in the center of the colon specimen using an 18‐G injection needle (Terumo Co., Tokyo, Japan) and a 2 mm biopsy punch (KAI Medical Co., Gifu, Japan). After insertion of the catheter into the lumen, the ends of the colonic specimen were ligated, and the catheter was connected to an infusion pump and a blood pressure transducer (ADInstruments Japan, Nagoya, Japan). Either VC or saline was infused at 1.5 mL/min. The maximum intraluminal pressure prior to leakage was recorded as the burst pressure.

### 2.4 Acute Systemic Toxicity Test (ISO 10993‐11:2017) ^18^

VC or saline was administered intraperitoneally to 10 rats (*n* = 5 per group) as a single dose of 10 or 20 mL/kg. For all animals, the general condition and body weight were evaluated at 24, 48, and 72 h after the administration of VC or saline. Necropsy for macroscopic examination was performed after the observation period. All animals were euthanized by exsanguination under deep anesthesia induced with sodium pentobarbital. The external surface, including the injection site, was examined macroscopically. After laparotomy, the abdominal cavity was inspected for hemorrhage, fluid accumulation, abscesses, or adhesions. Organs (brain, pituitary, thyroid, heart, thymus, aorta, trachea, bronchi, lungs, liver, kidneys, adrenals, spleen, pancreas, stomach, intestines, testes, epididymides, prostate, and seminal vesicles) were excised intact, examined for size, color, shape, consistency, and appearance, and abnormalities were recorded. If no animal in the VC group exhibited stronger reactivity than the animals in the saline group, VC was judged to have no acute systemic toxicity. Additionally, blood samples were collected on days 4, 7, and 14 of treatment from another 18 rats (*n* = 3 per group) that were similarly administered VC, and the hematology and blood biochemistry parameters were measured and compared with in‐house historical normal rat control data.

### 2.5 Bacterial Growth Test

*Escherichia coli* (*Escherichia coli* ATCC 25922; ATCC, Manassas, VA, USA) was grown on soybean–casein digest agar at 35°C under aerobic conditions. Colonies were suspended in distilled water to prepare bacterial suspension no. 1 using the McFarland standard (bioMérieux SA, Marcy l'Etoile, France), and then diluted at 1:10,000 with 5× Mueller Hinton II Broth Cation‐adjusted (Becton, Dickinson and Company, USA) to prepare bacterial suspension no. 2. Twelve sterilized test tubes were prepared. Sixty microliters of suspension no. 1 and 3 mL of the test material (VC or saline, *n* = 3 per group) were added to each test tube. Similarly, 600 µL of bacterial suspension no. 2 and 2.4 mL of each test material were added to each test tube. All reaction mixtures were incubated under aerobic conditions at 35°C for 6 and 24 h. Viable bacteria in the serially diluted fluids were counted by the pour plate method using AccuDia Trypto‐Soya Agar (Shimadzu Diagnostics Corporation, Tokyo, Japan). The detection limit of the viable bacteria was 10 colony‐forming units (CFUs). The CFU/mL was calculated as follows and converted to logarithmic values: CFU/mL = viable bacterial count × 10 × dilution ratio. The bacterial growth rates (μ) at 6 and 24 h of incubation were calculated as follows: μ(t_0–6_) = [log_10_ N(t6)—log_10_ N(t0)] / 6, and μ(t_0–24_) = [log_10_ N(t24)—log_10_ N(t0)] / 24, where *N* represents CFU/mL.

### 2.6 Cytotoxicity Test (ISO 10993–5:2009) ^18^

V79 cells (passage number 9; Health Science Research Resources Bank) were seeded at 0.5 × 10^2^ cells/mL in 6‐well plates (2.0 mL/well). A total of four plates (*n* = 3 wells per group) were prepared. After 24 h of incubation, VC was added at final concentrations of 0.31 to 10.0 v/v% and incubated in a CO_2_ incubator (5% CO_2_, 37°C) for 6 days. After staining with Giemsa, the numbers of colonies were counted. The relative colony‐forming rate of the V79 cells was determined and compared with the control value, and the half‐maximal inhibitory concentration was calculated from the relative cell growth rate.

### 2.7 Sensitization Test (ISO 10993‐10:2010) ^18^

In the first induction phase, a water‐in‐oil type emulsion of saline and Freund's complete adjuvant, VC alone, and a water‐in‐oil type emulsion of VC and Freund's complete adjuvant were administered intradermally into the intrascapular region of 15 guinea pigs (*n* = 5 per group, 0.1 mL/site). In the second induction phase, 10 w/w% sodium lauryl sulfate was applied topically to the intrascapular region 5 days later. After 24 h, filter paper patches saturated with 0.2 mL of VC or saline were applied in an occlusive manner and left for 48 h. For the challenge phase (21 days after the first induction), two lint patches saturated with 0.1 mL of VC or saline were applied in an occlusive manner to the abdominal and dorsal flanks of each guinea pig for 24 h. The skin reaction at each challenge site was graded numerically using the Magnusson and Kligman scale at 24 and 48 h after patch removal.

### 2.8 Intracutaneous Irritation Test (ISO 10993‐10:2010) ^18^

VC and saline were injected once intracutaneously into the back of each of 3 rabbits (0.2 mL/injection). VC was injected at five sites to the left of the spine, while saline was injected at five sites to the right of the spine. Each injection site was observed macroscopically at 24, 48, and 72 h after injection and graded numerically for erythema/eschar and edema using the grading system for intracutaneous (intradermal) reactions. The final score was calculated in accordance with the ISO 10993‐10:2010 guidelines; if the score was 1.0 or less, the VC gel was judged “acceptable” in the intradermal reactivity test.

### 2.9 Data Collection

Experimental parameters were assessed by trained evaluators. Blinding of evaluators to group allocation was not performed in any of the trials in this study.

### 2.10 Statistical Analysis

Data are presented as the mean ± standard deviation or 95% confidence interval of the mean. Statistical significance was assessed with the unpaired t‐test using SAS 9.4 software (SAS Institute, Cary, NC, USA). The significance level was set at *p* < 0.05.

## 3 Results

### 3.1 Colonic Burst Pressure

**FIGURE 2:**
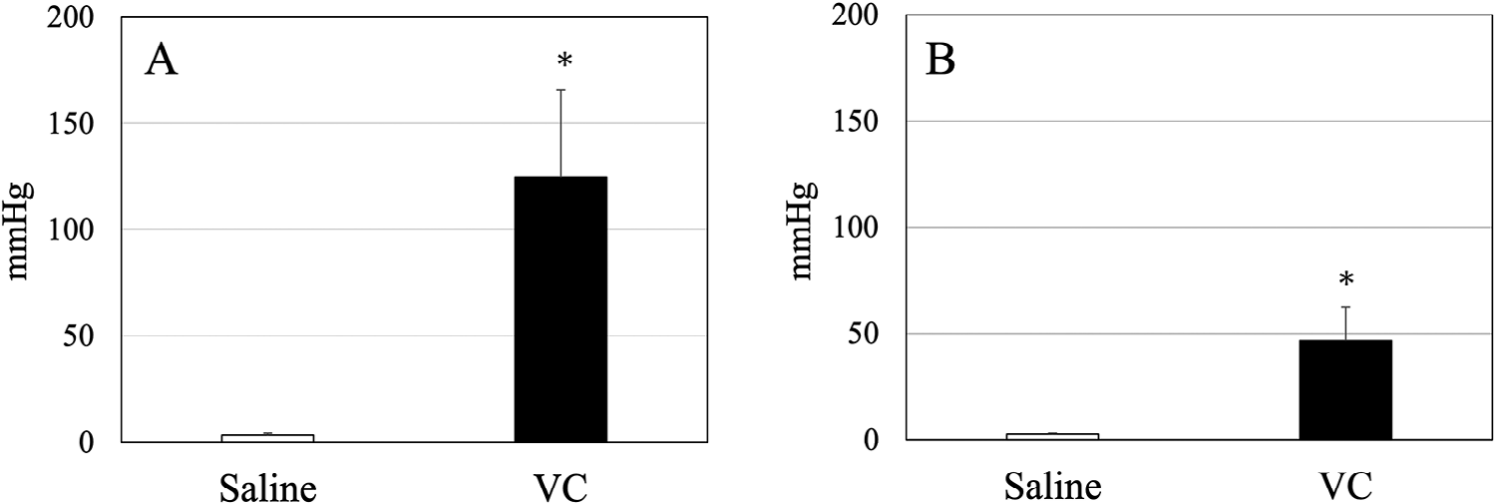
Colonic burst pressure after infusion of VC or saline. A full‐thickness defect was created using (A) an 18‐G injection needle and (B) a 2 mm biopsy punch. Data are presented as mean ± standard deviation (*n* = 5). *p < 0.05. VC, Viscoclear.

The colonic burst pressure was significantly higher for VC compared with saline under both the 18‐G and 2‐mm full‐thickness defect conditions (Figure 2).

### 3.2 Acute Systemic Toxicity

After the intraperitoneal injections, there were no changes in the general condition of any animal, and the body weights were similar in the VC and saline groups. There were also no abnormalities, including accumulation of abdominal fluid, intra‐abdominal abscess or adhesion formation, at necropsy performed after the observation periods (Table 1), and no abnormal hematological and blood biochemical test results, even in the high‐dose VC group (Table 2).

**TABLE 1:** Acute systemic toxicity test of Viscoclear (VC) in rats.

	20 mg/kg of VC				Saline			
	4 h	24 h	48 h	72 h	4 h	24 h	48 h	72 h
General condition	NA	NA	NA	NA	NA	NA	NA	NA
Body weight, g; mean (standard deviation)	155 ± 4	158 ± 3	167 ± 4	179 ± 3	155 ± 4	163 ± 4	173 ± 5	184 ± 6
Necropsy findings^†^	No data			NA	No data			NA
abdominal fluid intra‐abdominal abscess adhesion formation								

g, grams; NA: no abnormalities; VC, Viscoclear

^†^ Macroscopic examination was performed on the following organs and intra‐abdominal findings: the injection site, brain, pituitary gland, thyroid gland, heart, thymus, aorta, trachea, bronchi, lungs, liver, kidneys, adrenal glands, spleen, pancreas, stomach, duodenum, jejunum, ileum, cecum, colon, rectum, testes, epididymides, prostate, and seminal vesicles.

**TABLE 2:** Hematology and blood chemistry of rats intraperitoneally injected with Viscoclear (VC).

	Control^†^ (lower ‐ upper)			10 mg/kg of VC									20 mg/kg of VC								
				Day 4			Day 7			Day 14			Day 4			Day 7			Day 14		
Hematology																					
WBC (10^2^/µL)	29.9	—	168.7	123.4	±	16.4	146.3	±	6.3	112.9	±	19.8	138.9	±	12.4	157.4	±	13.2	148.0	±	27.0
RBC (10^4^/µL)	534	—	754	616	±	11	644	±	24	704	±	41	609	±	6	632	±	23	683	±	33
HGB (g/dL)	11.6	—	15.2	13.3	±	0.2	13.5	±	0.5	14.5	±	0.7	13.1	±	0.2	13.4	±	0.3	14.4	±	0.3
HCT (%)	35.5	—	43.1	39.2	±	0.8	39.4	±	1.3	41.2	±	1.5	38.9	±	0.9	39.5	±	0.3	41.2	±	0.8
PLT (10^4^/µL)	68.7	—	155.9	148.3	±	17.3	124.9	±	18.2	108.8	±	8.9	146.0	±	0.9	143.7	±	21.1	116.6	±	8.7
NEUT%	0.2	—	32.6	14.1	±	5.5	13.9	±	6.2	13.0	±	1.8	13.2	±	4.8	15.8	±	5.1	16.1	±	0.9
LYMPH%	61.2	—	96.8	82.2	±	5.9	82.1	±	4.9	82.4	±	1.6	83.5	±	5.7	80.2	±	5.3	80.5	±	1.6
MONO%	1.2	—	6.4	2.6	±	0.5	2.8	±	1.1	3.7	±	0.7	2.2	±	0.8	2.8	±	0.6	2.6	±	0.6
Blood chemistry																					
AST (U/L)	58	—	146	89	±	29	87	±	17	91	±	8	76	±	10	81	±	11	79	±	12
ALT (U/L)	20	—	52	33	±	4	34	±	3	33	±	3	37	±	13	39	±	8	28	±	5
ALP (U/L)	162	—	1710	780	±	133	690	±	33	725	±	51	942	±	155	698	±	104	837	±	64
T‐P (g/dL)	4.61	—	5.69	5.5	±	0.2	5.3	±	0.2	5.7	±	0.2	5.3	±	0.2	5.3	±	0.1	5.5	±	0.1
ALB (g/dL)	2.44	—	3.48	3.1	±	0.2	3.1	±	0.1	3.2	±	0.2	3.1	±	0.1	3.1	±	0.1	3.2	±	0.1
A/G	0.95	—	1.79	1.3	±	0.1	1.4	±	0.1	1.3	±	0.1	1.4	±	0.1	1.4	±	0.1	1.4	±	0.1
GLU (mg/dL)	68	—	184	130	±	5	141	±	29	137	±	13	125	±	21	128	±	19	130	±	12
TG (mg/dL)	4	—	112	55	±	8	52	±	11	51	±	6	53	±	11	32	±	3	48	±	14
T‐CHO (mg/dL)	46	—	94	80	±	6	84	±	13	59	±	6	66	±	13	77	±	14	60	±	8
PL (mg/dL)	78	—	142	127	±	11	126	±	10	102	±	7	107	±	14	112	±	11	103	±	7
T‐BIL (mg/dL)	0.02	—	0.10	0.06	±	0.02	0.07	±	0.02	0.06	±	0.00	0.06	±	0.01	0.05	±	0.01	0.06	±	0.01
UN (mg/dL)	10.7	—	27.5	24.8	±	4.2	23.4	±	4.8	23.5	±	5	23	±	1.7	25.8	±	8.3	22.2	±	5.9
CRE (mg/dL)	0.15	—	0.31	0.22	±	0.02	0.18	±	0.03	0.22	±	0.05	0.20	±	0.04	0.22	±	0.02	0.25	±	0.02

Data are presented as mean ± standard deviation (*n* = 3).

^†^ Values represent a 95% confidence interval of the mean of in‐house historical normal rat control data (*n* = 35).

### 3.3 Bacterial Growth

**FIGURE 3:**
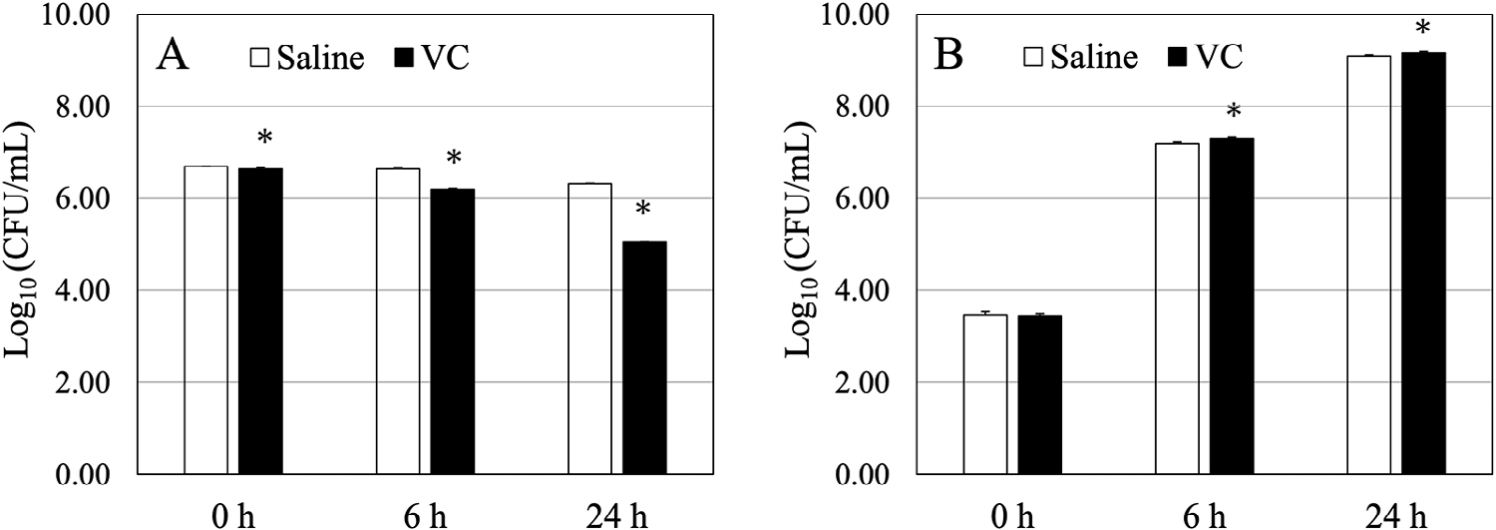
Bacteria growth in VC or saline. Viable *Escherichia coli* counts under (A) nutrient‐free and (B) nutrient‐rich conditions. Data are presented as mean ± standard deviation (*n* = 3). *p < 0.05. VC, Viscoclear.

Under nutrient‐free conditions, VC inhibited bacterial growth compared with saline, resulting in a 20‐fold reduction in viable bacterial counts after 24 h of incubation (Figure 3). The bacterial growth rates differed between the groups (*p* < 0.01, μ(t_0–6_): ‐0.010 vs. ‐0.074, μ(t_0–24_): ‐0.016 vs. ‐0.066). In contrast, under nutrient‐rich conditions, the viable bacterial count was 1.2 times higher in the VC group than in the saline group after 24 h of incubation (*p* < 0.05). However, there were no significant differences in bacterial growth rates between the groups (μ(t_0–6_): 0.619 vs. 0.640, μ(t_0–24_): 0.234 vs. 0.238).

### 3.4 Cytotoxicity

VC showed no cytotoxicity that inhibited the colony stimulation rate of V79 cells (Table 3).

**TABLE 3:** Viscoclear (VC) cytotoxicity test in V79 cells.

	Final conc.	Number of colonies			Relative colony‐forming rate (%)	IC_50_ (%)
Water (vehicle control)	0 v/v%	104.7±12.1			100.0	Not calculated
VC	0.31 v/v%	110.7±5.8			105.7	Not calculated
	0.63 v/v%	105.0±3.6			100.3	
	1.3 v/v%	107.3±10.1			102.5	
	2.5 v/v%	95.0±7.5			90.7	
	5.0 v/v%	99.0±7.5			94.6	
	10.0 v/v%	105.7±4.0			101.0	

Data are presented as mean ± standard deviation (*n* = 3). IC_50_, half maximal inhibitory concentration; VC, Viscoclear The validity of this test was confirmed using a zinc dibutyl dithiocarbamate (positive control).

### 3.5 Sensitization

There were no changes in the skin appearance at the VC challenge sites, and the gel showed no skin sensitizing potency (Table 4).

**TABLE 4:** Viscoclear (VC) skin sensitization test in guinea pigs.

	Animal No.	Challenge site at 24 h		Challenge site at 48 h	
		VC	saline	VC	Saline
Negative control	1, 2, 3, 4, 5	0, 0, 0, 0, 0	0, 0, 0, 0, 0	0, 0, 0, 0, 0	0, 0, 0, 0, 0
VC	6, 7, 8, 9, 10 11, 12, 13, 14, 15	0, 0, 0, 0, 0 0, 0, 0, 0, 0	0, 0, 0, 0, 0 0, 0, 0, 0, 0	0, 0, 0, 0, 0 0, 0, 0, 0, 0	0, 0, 0, 0, 0 0, 0, 0, 0, 0

VC, Viscoclear. Skin reaction at each challenge site was graded in accordance with the Magnusson and Kligman scale. The validity of this test was confirmed using 0.1% 1‐chloro‐2,4‐dinitrobenzene (positive control).

### 3.6 Intracutaneous Irritation

All injection sites showed erythema and very mild irritation, but this was judged “acceptable” because the final score of the macroscopic observations was less than 1.0 (Table 5). There was also a white bulge at the injection sites that diminished over time and was determined to be due to residual VC.

**TABLE 5:** Viscoclear (VC) intracutaneous reactivity test in rabbits.

	VC			Saline		
Observation timepoint	24 h	48 h	72 h	24 h	48 h	72 h
Erythema and eschar formation^†^	5, 5, 5	5, 5, 5	5, 5, 5	0, 0, 0	0, 0, 0	0, 0, 0
Edema^†^	0, 0, 0	0, 0, 0	0, 0, 0	0, 0, 0	0, 0, 0	0, 0, 0
Final score	1.0					

^†^ Sum of the grades for erythema/eschar and edema observed in each animal (five sites/animal, *n* = 3) The final score was calculated using the ISO 10993‐10:2010 guidelines, and a score of 1.0 or less was judged “acceptable”.

## 4 Discussion

Given the possibility that accidental gastrointestinal perforation during gel immersion EMR or ESD could result in leakage of VC from the lumen into the peritoneal cavity, we evaluated the safety of VC by mimicking such circumstances. The present results demonstrated that VC has a lower propensity to leak from the lumen compared with saline, and VC leakage into the peritoneal cavity caused no acute systemic reactions in animal experiments. Furthermore, VC did not promote the growth of *E. coli* to a greater extent than saline under nutrient‐free conditions. Although the VC group had a significantly higher viable bacterial count than the saline group under nutrient‐rich conditions, the actual difference between groups was small, and there was no intergroup difference in bacterial growth rates. Finally, VC caused no cytotoxicity, sensitization, or irritation, indicating that VC is a highly biocompatible material.

Gastrointestinal perforations that occur during EMR or ESD are most commonly small and are typically managed with secure endoscopic clipping and conservative treatment [16, 17]. Using an ex vivo model that mimicked small perforations, we observed a higher colonic burst pressure in the VC group, indicating that VC was less likely to leak from the lumen than saline. This finding suggests that gel immersion endoscopy may confer a safety advantage over SITE.

The safety assessment after an intraperitoneal administration of VC at a high dose of 20 mL/kg revealed no abnormalities in the general condition, necropsy findings, and hematological or biochemical parameters of rats. This dose corresponds to approximately 1000 mL of gel in a 50‐kg human, which is considerably greater than the actual volume of VC expected to leak in clinical practice, indicating that VC has a wide safety margin. Moreover, the total amount of polysaccharide components (xanthan gum and locust bean gum) in VC is only 0.15 g/200 g/package, indicating that the absolute quantity of leaked gel during clinical use would be minimal. VC resulted in no adverse effects in cytotoxicity, sensitization, or irritation studies, further demonstrating the high biocompatibility of VC. In addition, reports suggest that xanthan gum has an inhibitory effect on the production of inflammatory cytokines [19], implying that minor leakage of VC gel is unlikely to induce excessive inflammation.

The present study has several limitations. First, we did not investigate the distribution, metabolism, or excretion of VC after its absorption into the body. Second, although VC injected into the intestinal lumen comes into contact with enteric bacteria, and both VC and bacteria may leak into the peritoneal cavity after perforation, we did not assess the safety of intraperitoneal administration of a mixture of VC and bacteria. However, in in vitro culture experiments under nutrient‐free conditions, VC reduced the growth of *E. coli* by 20‐fold compared with saline. In contrast, under nutrient‐rich conditions, although there was a statistically significant difference in the mean value between the groups, the magnitude of this difference was small (only 1.2‐fold). Furthermore, the biologically relevant bacterial growth rates were comparable. These results suggest that VC does not serve as a substrate for *E. coli* growth, and that the risk of infection is comparable for EMR and ESD when using either gel or saline. However, as the bacterial culture experiments in this study were limited to *E. coli*, caution is required when extrapolating these findings to clinical situations, where perforations typically involve polymicrobial communities dominated by anaerobic species such as Bacteroides. Third, we did not conduct a safety evaluation using large animal models. Large animal models are capable of reproducing complex physiological responses, such as human‐like immune and metabolic functions, that may not be adequately replicated in small animal models. Fourth, although every effort was made to minimize bias in the study design, data collection, and interpretation, we acknowledge that the affiliation of five of the six authors with the manufacturing company and the fact that the study was funded by this company could represent a potential source of optimistic bias. Readers should interpret the findings with this context in mind. Fifth, as blinding of the evaluators to group allocation was not implemented in this study, the possibility that observer bias may have influenced the assessments cannot be excluded. This is a limitation of the study and should be taken into consideration when interpreting the findings. To overcome these limitations and further enhance the clinical relevance of the present findings, future studies should first evaluate the effects of VC on additional bacterial species, including anaerobic organisms such as Bacteroides. Second, long‐term assessments of biological responses should be conducted using in vivo perforation or peritonitis models in large animals. Integrating the outcomes of these future investigations with the current results will provide a more comprehensive understanding of the safety and biocompatibility of VC.

In summary, VC demonstrates a lower risk of leakage from the lumen. Even if a small amount were to leak into the peritoneal cavity, the experimental results demonstrating no acute systemic toxicity and acceptable biocompatibility suggest that VC has a safety profile comparable to that of saline. However, comprehensive risk assessment through long‐term safety studies remains warranted. Clinically, when a perforation occurs during gel immersion EMR or ESD using VC, a minimal amount of additional VC should be used during closure to maintain low intraluminal pressure. Prompt aspiration after closure would minimize peritoneal exposure and align with the demonstrated safety margin.

## Author Contributions

Atsushi Ohata and Tomonori Yano designed the study, the main conceptual ideas, and wrote the draft manuscript. Atsushi Ohata, Ryo Kawahara, Yuji Hiraki, Hikaru Nakata, and Shinya Kaneda collected the data. Atsushi Ohata, Ryo Kawahara, Yuji Hiraki, and Hikaru Nakata aided in interpreting the results and worked on the manuscript. Tomonori Yano supervised the project. Atsushi Ohata wrote the manuscript with support from Tomonori Yano. All authors discussed the results and commented on the manuscript.

## Conflicts of Interest

Tomonori Yano and Atsushi Ohata hold patents for and are the inventors of the dedicated gel for the gel immersion method. Five of six authors (Atsushi Ohata, Ryo Kawahara, Yuji Hiraki, Hikaru Nakata, and Shinya Kaneda) are employees of Otsuka Pharmaceutical Factory, Inc., the company that manufactures Viscoclear.

## Funding

This study was funded by Otsuka Pharmaceutical Factory, Inc.

## Ethics Statement

None.

## Consent

N/A.

## Clinical Trial Registration

N/A.

## Data Availability

The data that support the findings of this study are available from the corresponding authors (Tomonori Yano and Atsushi Ohata) upon reasonable request.
